# Prevalence of SARS-CoV-2 antibodies among Swiss hospital workers: Results of a prospective cohort study

**DOI:** 10.1017/ice.2020.1244

**Published:** 2020-10-08

**Authors:** Philipp P. Kohler, Christian R. Kahlert, Johannes Sumer, Domenica Flury, Sabine Güsewell, Onicio B. Leal-Neto, Julia Notter, Werner C. Albrich, Baharak Babouee Flury, Allison McGeer, Stefan Kuster, Lorenz Risch, Matthias Schlegel, Pietro Vernazza

**Affiliations:** 1 Cantonal Hospital St Gallen, Division of Infectious Diseases and Hospital Epidemiology, St Gallen, Switzerland; 2 Children’s Hospital of Eastern Switzerland, Department of Infectious Diseases and Hospital Epidemiology, St Gallen, Switzerland; 3 Clinical Trials Unit, Cantonal Hospital of St Gallen, St Gallen, Switzerland; 4 Epitrack, Recife, Brazil; 5 Department of Economics, University of Zurich, Zurich, Switzerland; 6 Sinai Health System, Toronto, Ontario, Canada; 7 Federal Office of Public Health, Berne, Switzerland; 8 Labormedizinisches Zentrum Dr. Risch, Buchs, Switzerland

## Abstract

In this prospective cohort of 1,012 Swiss hospital employees, 3 different assays were used to screen serum for SARS-CoV-2 antibodies. Seropositivity was 1%; the positive predictive values of the lateral-flow immunoassay were 64% (IgG) and 13% (IgM). History of fever and myalgia most effectively differentiated seropositive and seronegative participants.

Coronavirus disease 2019 (COVID-19) is currently threatening global health. Evidence from various countries indicates that healthcare workers (HCWs) are at increased risk for COVID-19.^1,2^ Many previous studies have focused on the molecular testing of symptomatic HCWs, ignoring the fact that a significant proportion of infected people might not exhibit any symptoms and that false-negative PCR results are not uncommon.^3,4^ Although available serologic tests have somewhat uncertain performance characteristics, assessment of antibodies to severe acute respiratory virus-2 (SARS-CoV-2) provides a better estimate of the true prevalence and has therefore been recommended by leading healthcare experts.^5^

The aims of this prospective cohort study were to assess seropositivity for SARS-CoV-2, to identify risk exposures, and to describe the spectrum of COVID-19 symptoms among hospital workers.

## Methods

### Participants and setting

Between March 19 and April 3, 2020, hospital workers (≥16 years) from 2 tertiary-care hospitals (Cantonal Hospital St Gallen and the Children’s Hospital of Eastern Switzerland) were invited to participate. COVID-19 cases in this region peaked between March 23 and March 30 (53 cases per 100,000 population per week). Hospital admissions were highest in the second week of April.

### Serology testing

Participants’ sera were analyzed for SARS-CoV-2 antibodies using 3 different tests: a lateral flow immunochromatographic assay (LFIA, Sugentech, Yuseong-gu, Daejeon, Republic of Korea), a chemiluminescence microparticle immunoassay (CMIA, Abbott Diagnostics, Lake Bluff, IL), and an electro-chemiluminescence immunoassay (ECLIA, Roche Diagnostics, Basel, Switzerland). Participants with a positive signal in any test provided a second sample 4 weeks later (all tests performed again). True seropositivity was assumed in cases of positive IgG in LFIA and either CMIA or ECLIA (at the same time). This procedure corresponds to an orthogonal testing algorithm in which an independent second test confirms the positive result of the first test (https://www.cdc.gov/coronavirus/2019-ncov/lab/resources/antibody-tests-guidelines.html). Samples with positive IgG in LFIA only were additionally tested with a chemiluminescence immunoassay (CLIA) directed at the spike proteins S1/S2 (DiaSorin, Italy). PCR was not routinely performed.

### Online questionnaire

Participants filled in a web-based questionnaire asking about respiratory and general symptoms and COVID-19 exposures (3 weeks prior to baseline testing). The intensity of patient contact was stratified as follows: HCWs caring for confirmed COVID-19 patients; HCWs exposed to patients without known COVID-19; and others.

### Statistical analysis

Participants with positive or negative serology were compared. The Fisher exact test was used for dichotomous variables; the Mann-Whitney U test was used for continuous variables. The positive predictive value (PPV) was calculated for LFIA using the true positives as numerator and all positive test results as denominator (baseline and follow-up results were pooled). Analyses were performed with R version 3.6.3 software (R Foundation for Statistical Computing, Vienna, Austria). The Ethical Commission of Eastern Switzerland approved the study (no. 2020-00502).

## Results

We included 1,012 hospital workers with a median age of 38.3 years (range, 16.9–64.8); 761 (75%) were women. Most were nurses (n = 398, 39%) or physicians (n = 268, 27%) (Table 1).

**Table 1.** Baseline Characteristics of Study Participants

Characterstic	All (n=1,012), No. (%)	Seropositive (n=10), No. (%)	Seronegative (n=1,002), No. (%)	*P* Value^a^
**General**				
Age, median y (range)	38.3 (16.9–64.8)	33.4 (23.8–46.4)	38.5 (16.9–64.8)	.15
BMI, median kg/m^2^(range)	23.1 (16.6–47.0)	22.0 (19.4–35.5)	23.1 (16.6–47.0)	.74
Gender				
Male	251 (24.8)	2 (20)	249 (24.9)	1.00
Female	761 (75.2)	8 (80)	753 (75.1)	
Ethnicity				
European	991 (97.9)	9 (90)	982 (98.0)	.19
Other	21 (2.1)	1 (10)	20 (2.0)	
Work place				
Adult acute care	920 (90.9)	10 (100)	910 (90.8)	.77
Childrens hospital	92 (9.1)	0 (0)	92 (9.2)	
Profession				.62
Physician	268 (26.5)	4 (40)	264 (26.3)	
Nurse	398 (39.3)	3 (30)	395 (39.4)	
Other	346 (34.1)	3 (30)	343 (34.3)	
Smoking				
No	647 (63.9)	7 (70)	640 (63.9)	.79
Formerly	247 (24.4)	3 (30)	244 (24.4)	
Active	118 (11.7)	0 (0)	118 (11.8)	
Home office				
Yes	16 (1.6)	0 (0)	16 (1.6)	1.00
Partly	123 (12.2)	1 (10)	122 (12.2)	
No	873 (86.3)	9 (90)	864 (86.2)	
Time of baseline testing				.98
Week 1	604 (59.7)	6 (60)	598 (59.7)	
Week 2	408 (40.3)	4 (40)	404 (40.3)	
**Nonwork Exposure**				
Daily time in public transport				
0 min	599 (59.2)	6 (60)	593 (59.2)	.95
1–15 min	152 (15.0)	2 (20)	150 (15.0)	
16–30 min	122 (12.1)	1 (10)	121 (12.1)	
>30 min	139 (13.7)	1 (10)	138 (13.8)	
Symptomatic person in household				
No	834 (82.4)	6 (60)	828 (82.6)	.08
Yes	178 (17.6)	4 (40)	174 (17.4)	
COVID-19 case in household				
No	1,011 (99.9)	9 (90)	1,002 (100)	**.010**
Yes	1 (0.1)	1 (10)	0	
Unprotected contact with confirmed COVID-19 cases outside of work or household				
No	980 (96.8)	8 (80)	972 (97.0)	**.04**
Yes	32 (3.2)	2 (20)	30 (3.0)	
Unprotected contact with other symptomatic persons outside of work or household				
No	739 (73.0)	5 (50)	734 (73.3)	.14
Yes	273 (27.0)	5 (50)	268 (26.7)	
**Work Exposure**				
Contacts with COVID-19–positive coworker				
Never	683 (67.5)	7 (70)	676 (67.5)	.86
1–2 times	54 (5.3)	1 (10)	53 (5.3)	
3 and more times	23 (2.3)	0 (0)	23 (2.3)	
Do not know	252 (24.9)	2 (20)	250 (25.0)	
Intensity of patient contact				.20
HCW caring for known COVID-19 cases	209 (20.7)	3 (30)	206 (20.6)	
HCW caring for other patients	591 (58.4)	7 (70)	584 (58.3)	
Other hospital workers	212 (21.2)	0 (0)	212 (21.2)	
Wearing mask outside patient contact				
No	642 (63.4)	8 (80)	634 (63.3)	.34
Yes	370 (36.6)	2 (20)	368 (36.7)	
**Any patient contact (n=799)**				
Direct physical contact				
No	114 (14.3)	2 (20)	112 (14.2)	.64
Yes	685 (85.7)	8 (80)	677 (85.7)	
Involved in aerosol-generating procedures				
No	530 (66.3)	9 (90)	521 (65.9)	.18
Yes	269 (33.7)	1 (10)	268 (33.9)^b^	
Wearing a mask during patient contact				
No	234 (23.1)	7 (70)	227 (28.7)	**.009**
Yes	565 (55.8)	3 (30)	562 (71.1)^b^	
**Contact with COVID-19 patients (n=209)**				
Close COVID-19 patient contact				
No	108 (51.7)	2 (66.7)	106 (51.5)	1.00
Yes	101 (48.3)	1 (33.3)	100 (48.5)	
Direct droplet exposure				
No	177 (84.7)	3 (100)	174 (84.5)	1.00
Yes	32 (15.3)	0 (0)	32 (15.5)	

Note. HCW, healthcare worker.

a Wilcoxon rank sum test or the Fisher exact test.

b One participant answer not available.

**Fig. 1.**
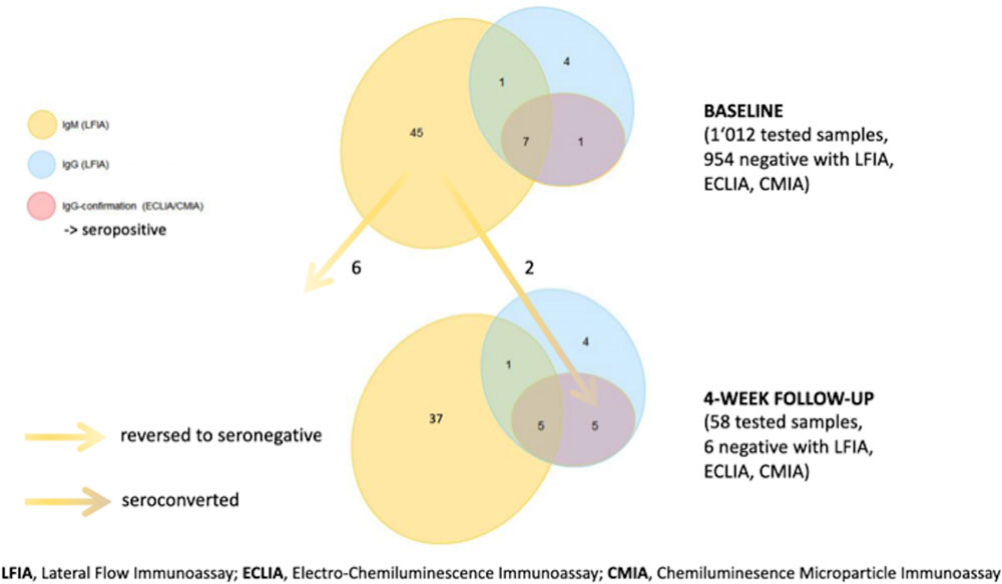
Results from baseline serology testing (n = 1,012) and from 4-week follow-up (n = 58). Cases with discrepant results between baseline and follow-up are highlighted with arrows.

At the baseline, 58 of 1,012 participants (5.7%) showed a positive signal in at least 1 test. In the LFIA, 13 participants had IgG (8 confirmed by ECLIA/CMIA) and 45 had IgM only. At follow-up, 2 participants showed a positive LFIA (IgG) and ECLIA/CMIA result in addition to the 8 samples confirmed at baseline, resulting in 10 of 1,012 true seropositives (1.0%) and 48 of 1,012 false seropositives (4.7%) (Fig. 1). Also, 5 participants had isolated LFIA IgG at baseline and follow-up and remained negative with anti-S1/S2 CLIA (DiaSorin). Overall, the PPV of the LFIA was 64% (18 true positive of 28 positive results) for IgG and 13% (12 true positive of 96 positive results) for IgM. ECLIA and CMIA results were consistent except for 1 participant (ECLIA negative and CMIA positive).

Seropositive participants more frequently reported fever or feverishness and limb or muscle pain than seronegative participants. Respiratory symptoms were reported by 73% of seropositive and 54% of seronegative participants (*P* = .36). Similar differences were detected between participants with true-positive and false-positive serology results (Supplementary Figs. S1 and S2). Of the 10 seropositive patients, 2 (20%) denied any symptoms.

All positive participants worked in adult care (*P* = .77); 2 of 20 (10%) reported unprotected contact with confirmed COVID-19 cases outside work and household versus 30 of 972 (3%) among seronegative workers (*P* = .04). The intensity of patient contact was not associated with seropositivity (*P* = .20), although all seropositive participants reported some form of patient contact. Among those with patient contact, HCWs who wear masks were less likely to test positive (2 of 370, 0.5%) than those who did not wear masks (8 of 642, 1.2%) (*P* = .009) (Table 1).

## Discussion

In this prospective hospital worker cohort, 1% had SARS-CoV-2 antibodies detected at baseline. The high proportion of false-positive LFIA results (particularly IgM) underscores the low PPV of these tests when prevalence is low. Fever or feverishness and muscle or limb pain were most useful in discriminating patients with positive and negative serologies. The strengths of the study are the use of 3 different tests and our analysis of a followup sample in case of a positive signal at baseline.

Given the COVID-19 peak in the region around collection of baseline samples, and considering a latency between infection and IgG detection of 2–3 weeks, these data represent an early phase of the local epidemic. This explains the low positivity rate and the low PPV of 64% (IgG) and 13% (IgM) for the LFIA. Data suggest cross reactions between endemic coronaviruses and SARSCoV-2, particularly for assays targeting the nucleocapsid protein.^6^ However, despite being directed against nucleocapsid, the CMIA and ECLIA used in this study have previously demonstrated excellent specificity (>99%) and acceptable sensitivities (93.9 and 87.7%, respectively).^7^ The concordant results between CMIA and ECLIA, as well as between CMIA/ECLIA and CLIA (directed at S1/S2 proteins), further strengthen our confidence in the specificity of the tests. Regarding sensitivity, recent evidence shows that a humoral immune response is mounted less frequently in patients with mild COVID-19.^8^ Indeed, the reported sensitivities of the CMIA and ECLIA are 93.9 and 87%, which might have underestimated our seroprevalence.^7^ Notably, LFIA screening identified 2 patients by positive IgM but negative CMIA and ECLIA at baseline, who eventually had IgG seroconversion in all tests. This finding is in line with data from the Infectious Diseases Society of America (IDSA) guideline on SARS-CoV serology testing showing a lower sensitivity of CMIA IgG compared to LFIA IgM early after infection.^9^

Although limited by the small case number, constitutional symptoms were more useful than respiratory symptoms in discriminating between seropositive and seronegative participants. Although COVID-19 may be a mild illness, it appears that illness caused by other respiratory viruses were more likely to cause isolated respiratory symptoms without constitutional symptoms than COVID-19. Furthermore, 20% of SARS-CoV-2–positive patients denied any symptoms, which is in line with current estimates.^3^

Serologically positive participants more likely reported exposure to COVID-19 cases outside work, whereas the intensity of patient contact did not differ compared to seronegative participants. Despite being compatible with data showing no increased seroprevalence of HCWs working in high-risk settings,^10^ these results should be interpreted with caution. Because of the early time point of baseline collection in relation to the local epidemic, providers might have had too little exposure to hospitalized COVID-19 patients to detect an exposure effect. The apparent protective effect of masks may reflect a reduction in undetected, unprotected exposure to other HCW or patients, or be an epi-phenomenon associated with adherence to other preventive practices.

In conclusion, seroprevalence was 1% at baseline in this prospective HCW cohort from Switzerland. Using 3 different tests, we challenge the usefulness of serology tests with limited specificity when prevalence is low. A prospective analysis of cohort data will allow us to better study the spectrum of symptoms and risk exposures associated with COVID-19.

## Supplementary material

For supplementary material accompanying this paper visit http://dx.doi.org/10.1017/ice.2020.1244. click here to view supplementary material
