# Supplementary material for: Prevalence of SARS-CoV-2 antibodies among Swiss hospital workers: Results of a prospective cohort study
Source: Infect Control Hosp Epidemiol. 2020 Oct 8:1–5. doi: 10.1017/ice.2020.1244 (PMC7582018; doi:10.1017/ice.2020.1244)

Supplementary Figures

Supplementary Figure 1. Symptoms of study participants within 3 weeks before baseline, by result of serology tests.


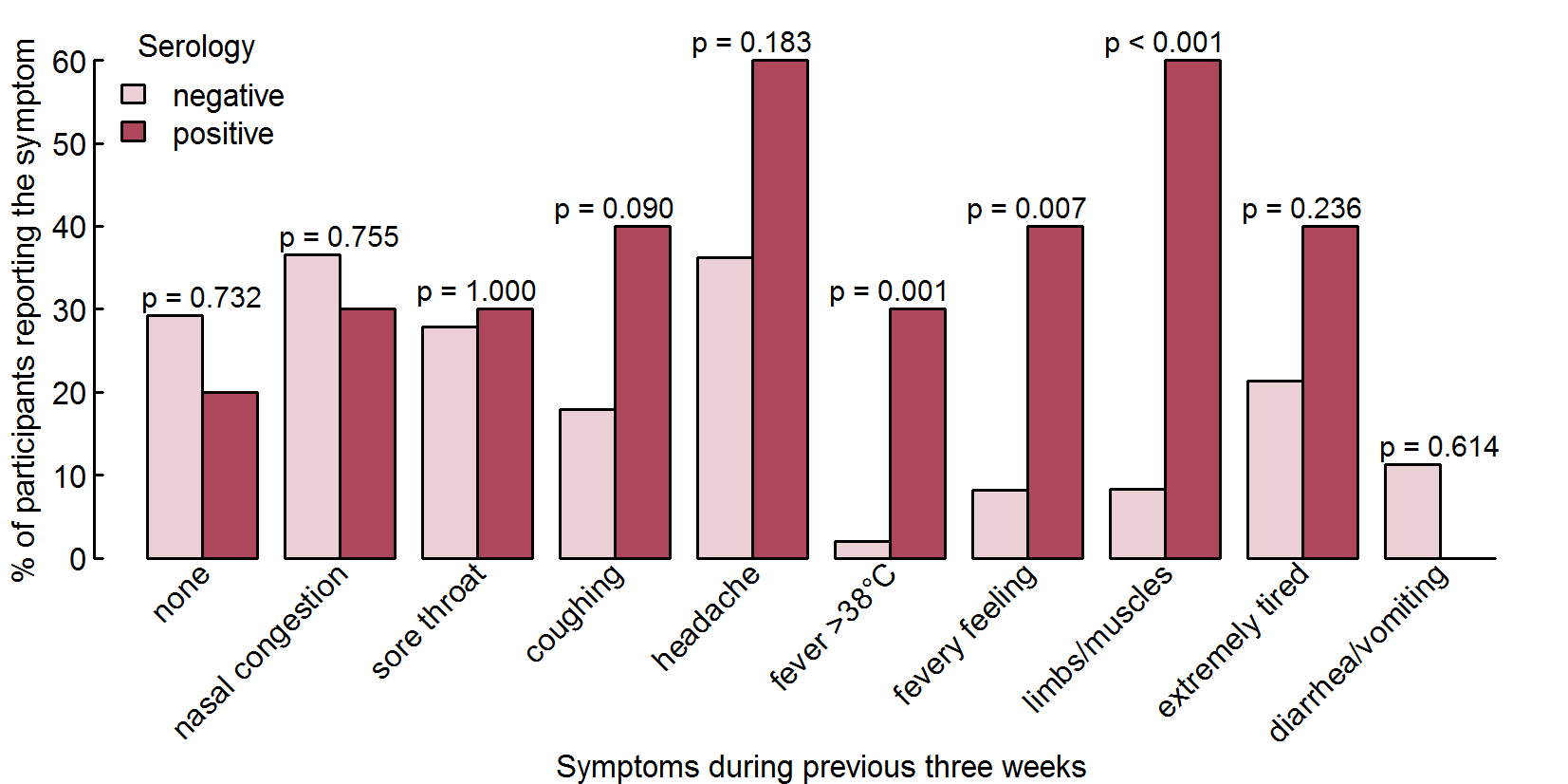


Supplementary Figure 2. Symptoms of participants with true and false positive serology within 3 weeks before baseline serology.


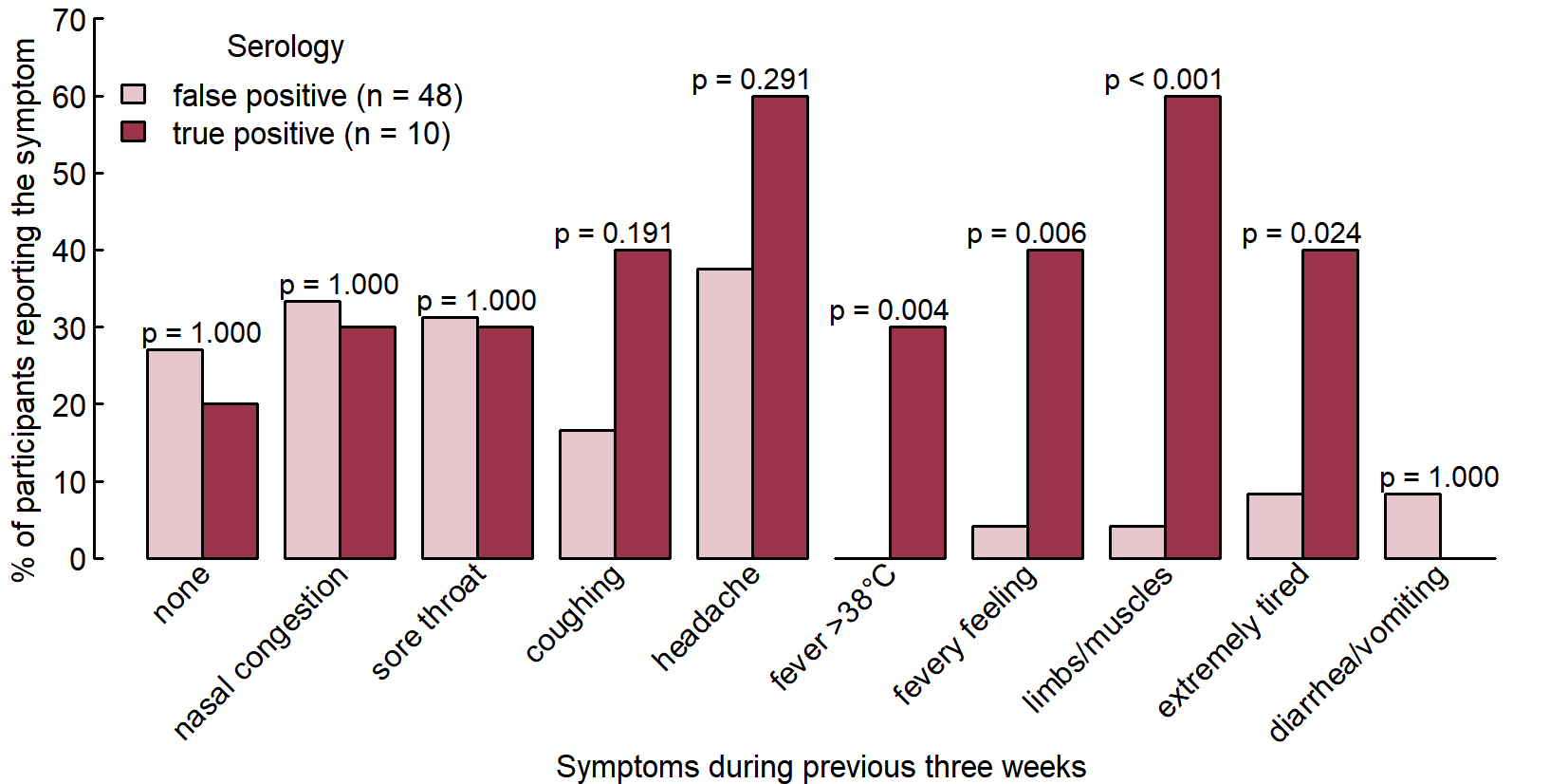

Supplement: Supplementary file 1 [file S0899823X20012441sup001.docx]
